# Primary myocardial involvement in systemic sclerosis: pathophysiology, clinical manifestations and advances in cardiac imaging

**DOI:** 10.1093/rheumatology/keag167

**Published:** 2026-04-04

**Authors:** Maya H Buch

**Affiliations:** Centre for Musculoskeletal Research, University of Manchester, Manchester, UK; NIHR Manchester Biomedical Research Centre, Manchester University Hospitals NHS Foundation Trust, Manchester, UK

**Keywords:** systemic sclerosis, primary heart, myocardial inflammation, myocardial fibrosis, cardiac MRI, echocardiography, autoimmune rheumatic disease

## Abstract

Systemic sclerosis (SSc) is a rare systemic autoimmune rheumatic disease associated with the highest cardiovascular risk among autoimmune conditions. Beyond pulmonary complications, primary heart involvement (pHI) represents a major cause of morbidity and mortality yet frequently remains asymptomatic and under-recognized. Microvascular dysfunction, myocardial inflammation and progressive interstitial and replacement fibrosis are central mechanisms. Clinical manifestations range from myocarditis to heart failure, arrhythmias and sudden cardiac death, while subclinical myocardial involvement is common and prognosis remains poor once overt disease develops. Advanced cardiovascular imaging, particularly cardiovascular magnetic resonance, has transformed detection through sensitive assessment of myocardial structure, function and myocardial tissue characterization, while PET may offer complementary insights. However, optimal screening strategies, imaging application and disease-specific therapies remain unmet needs. This review summarizes current understanding of SSc-pHI pathophysiology, clinical presentations and management, with a focus on the evolving role of multimodality imaging in early detection and risk stratification.

Rheumatology key messages

- Primary myocardial involvement in systemic sclerosis is common and frequently subclinical.
- Microvascular dysfunction, inflammation and fibrosis are central pathogenic mechanisms.
- Advanced cardiac imaging enables earlier detection and risk stratification.

## Introduction

Systemic sclerosis (SSc; also known as scleroderma) is a rare and complex systemic autoimmune rheumatic disease (SARD) characterized by the pathological triad of vasculopathy, immune activation and inflammation, and fibrosis (of the skin and internal organs) [1]. After pulmonary involvement [interstitial lung disease (ILD) and pulmonary arterial hypertension (PAH)], cardiovascular involvement is the next major cause of mortality, accounting for a third of all deaths [2]. A recent UK epidemiological study of 22 million individuals across 19 autoimmune diseases, including inflammatory arthritides and SARDs, identified SSc as being associated with the highest cardiovascular risk [3]. Cardiovascular involvement can be heterogeneous, spanning atherosclerotic cardiovascular disease to direct inflammatory myopericardial disease. The World Scleroderma Foundation/Heart Failure recommendations on the definition of SSc–primary heart involvement (pHI) emphasize the importance of distinguishing direct myocardial primary SSc heart involvement from secondary myocardial effects of other organ involvement (such as pulmonary hypertension and renal disease) as well as ischaemic (epicardial coronary) heart disease [4].

Although clinical manifestations may be heterogeneous, primary myocardial involvement in systemic sclerosis is often asymptomatic and subclinical, as demonstrated by numerous studies using sensitive cardiac imaging modalities, particularly cardiovascular magnetic resonance (CMR) [5–7]. Our understanding of SSc-pHI however remains relatively nascent, with major unmet needs. The exact mechanisms of SSc-pHI remain unclear; optimal use of CMR and other cardiovascular imaging for risk stratification and early detection and prognostic relevance needs systematic study. Also, no specific therapies for SSc-pHI exist [8].

This review focuses on SSc-pHI or primary myocardial involvement and details our current understanding of the pathophysiology, risk factors, clinical presentations and management. Given the emphasis of existing research on sensitive imaging approaches, this review examines their role in detecting and monitoring SSc-pHI.

## Mechanisms of myocardial involvement in systemic sclerosis

**Figure 1:**
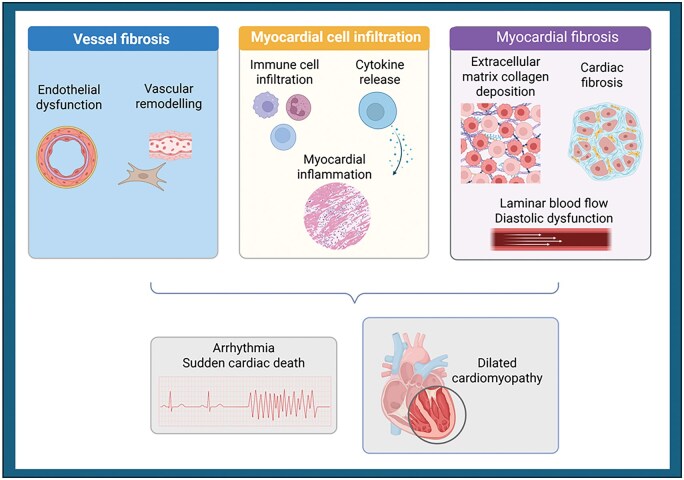
A schematic representation of the pathophysiology of primary heart involvement in systemic sclerosis. Primary heart involvement in systemic sclerosis results from three interrelated pathological processes: microvascular endothelial dysfunction and vascular remodelling, leading to myocardial vasculopathy and impaired myocardial perfusion; immune-mediated myocardial inflammation, driven by immune cell infiltration and cytokine release, resulting in myocardial injury and myocarditis; and progressive myocardial fibrosis, characterized by extracellular matrix collagen deposition, increased myocardial stiffness and diastolic dysfunction. These mechanisms give rise to clinical manifestations including myocarditis, arrhythmias with risk of sudden cardiac death, and heart failure with preserved ejection fraction. Created in BioRender. Buch, M. (2026) https://BioRender.com/7ilax0y

Histopathological studies provide key insights into the mechanisms underpinning SSc-pHI and support a central role for coronary microvascular dysfunction and myocardial infiltration, with both interstitial and replacement myocardial fibrosis described in SSc. An early, seminal autopsy series demonstrated focal myocardial lesions, including contraction band necrosis and replacement fibrosis consistent with ischaemia–reperfusion injury, distributed throughout both ventricles in the absence of obstructive epicardial coronary artery disease [9, 10]. Subsequent clinicopathological studies confirmed focal interstitial fibrosis with mild-to-moderate chronic inflammation as the predominant myocardial abnormality, with occasional myocyte dropout and contraction band necrosis occurring without associated inflammatory infiltrates [10, 11]. Importantly, myocardial involvement was frequently identified in patients without clinically overt cardiac disease. Collectively, these findings support a pathogenic model in which recurrent coronary microvascular ischaemia, driven by endothelial dysfunction and immune-mediated vascular injury that promote irreversible vessel wall fibrosis and remodelling of intramural coronary arteries, with or without myocardial inflammation leads to progressive interstitial and replacement fibrosis (Fig. 1). In keeping with this, coronary microvascular dysfunction, an established predictor of adverse cardiovascular outcomes in the absence of epicardial coronary disease [12], has been described in SSc [13].

Functional imaging studies also support a role for cardiac vasculopathy, sometimes referred to as ‘cardiac Raynaud’s phenomenon’ in SSc-pHI. Early single-photon emission computed tomography (SPECT) studies demonstrated reversible segmental perfusion defects in response to peripheral cold stimuli in the absence of coronary artery disease [14], consistent with vasospastic mechanisms. More recent CMR studies have reported a high prevalence of myocardial perfusion defects in SSc and demonstrated impaired myocardial blood flow responses following cold pressor testing [15].

Endomyocardial biopsy studies further highlight the fibrotic burden associated with SSc myocardial disease. In a retrospective analysis of 12 patients with biopsy-proven myocarditis, myocardial fibrosis was significantly more extensive in SSc compared with virus-negative myocarditis occurring in isolation or in other SARDs [16]. Interestingly, within the SSc cohort, fibrosis burden was similar in early and established disease and correlated with the extent of both cutaneous involvement and ventricular ectopy burden on ambulatory ECG monitoring.

Recent conceptual models propose a unified vascular phenotype in SSc, encompassing shared mechanisms of central and peripheral microvascular dysfunction [17]. However, the precise pathways linking myocardial involvement with systemic vasculopathy remain incompletely defined and need investigation.

## Epidemiology and risk factors

The exact prevalence of SSc-pHI specifically has been reported as ranging between 7% to >39% in clinical studies [18–20] and up to 80% in imaging-based studies and the earlier mentioned autopsy studies [9–11]. This wide-ranging variability reflects differences in definition of heart involvement (whether primary and secondary), cohort characteristics (symptomatic and asymptomatic) and detection method.

SSc-pHI may be asymptomatic, but once clinically apparent, portends a poor prognosis. An early single-centre study reported 60% survival rate 3 years from diagnosis [18]. In the EUSTAR cohort study of over 5000 patients, SSc-heart involvement accounted for 14% of overall deaths and non-SSc associated cardiovascular disease led to a further 12% of deaths [20]. Of note, trends in mortality from SSc, especially, cardiovascular involvement in SSc, has remained largely unchanged over 40 years [21].

A wide range of clinical factors have been associated with presence of SSc-pHI, including older age at time of diagnosis, diffuse cutaneous subset, autoantibody (Scl-70, RNA polymerase), skeletal muscle myopathy, ILD and complicated digital vasculopathy [22]. It is unclear whether each of these risk factors associates with different/specific manifestations of SSc-pHI (see below) and whether and how these may associate with each of the three principal pathogenic processes of SSc.

## Clinical manifestations

SSc-pHI can be characterized by myocarditis, heart failure (HF) and arrhythmia. However, subclinical involvement in asymptomatic individuals (of myocardial inflammation and early indicators of diastolic dysfunction) is common and well-recognized with the increasing use of advanced cardiovascular imaging, notably CMR [5–7], discussed later.

Consistent with the underlying pathophysiology, overt cardiac involvement in SSc is typically characterized by diastolic dysfunction, which may occur at any stage of the disease course and more commonly progresses to HF with preserved ejection fraction (HFpEF) rather than reduced ejection fraction [23]. Left ventricular diastolic dysfunction has been shown to independently predict increased mortality [24], further supporting early detection strategies.

SSc is also associated with increased arrhythmogenic risk, including sudden cardiac death and both atrioventricular and interventricular conduction abnormalities. Supraventricular and ventricular rhythm disturbances are described in SSc [25], ranging from premature ventricular complex including couplet and triplet beats, bigeminy, trigeminy, quadrigeminy, to more sustained arrhythmias such as ventricular tachycardia, and atrial fibrillation or flutter. However, reporting across studies is inconsistent in terms of definition (and method of detection) and often includes benign and non-specific findings on electrocardiogram (ECG). Moreover, electrophysiological abnormalities are often implicitly attributed to SSc-pHI, despite the potential contribution of co-existent ischaemic and/or other non-ischaemic aetiologies. Consequently, reported rates of SSc-pHI associated electrophysiological abnormalities vary widely (between 40% and 60% of cohorts), with likely over-estimation of the true frequency [26–28].

Nevertheless, arrhythmia remains an important sign of pHI to evaluate, with severe ventricular arrhythmia an ominous manifestation of SSc-HI and harbinger of poor outcome. Sudden cardiac death has been reported in up to 9% of individuals and is substantially more frequent among those with clinically overt pHI, with rates reported as high as 67% [10]. Vigilance and active monitoring are therefore essential. In addition, autonomic dysfunction is also a feature of SSc, with studies demonstrating higher heart rate, reduced heart rate variability and impaired heart rate recovery in individuals with SSc [29, 30].

Mechanistic understanding of electrophysiological abnormalities has not been investigated extensively to date. Although some studies have implicated myocardial fibrosis, particularly its burden, extent and involvement of the conduction system, as the pathological substrate for arrhythmias and conduction abnormalities, these associations remain incompletely defined. It is likely that microvasculopathy and inflammatory infiltration also contribute to arrhythmic risk. However, there has been limited molecular investigation of abnormal electrical signalling and excitation–contraction coupling, such as cardiomyocyte calcium and sodium handling to elucidate disease-specific mechanisms in SSc.

## Diagnostic evaluation and monitoring

### Role of cardiac imaging

#### Transthoracic echocardiography

Transthoracic echocardiography (TTE) is the most accessible and versatile imaging modality for the assessment of cardiac structure and function and is established in SSc management as a screening tool for PAH. TTE studies in SSc have commonly reported left ventricular diastolic dysfunction (rather than systolic dysfunction), ranging from 20% to up to 60% of cases [22]. Right ventricular changes have also been observed in the absence of PH [31, 32]. TTE, however, lacks sensitivity and specificity.

Speckled tracking echocardiography (STE) offers the potential to detect more subtle functional changes. STE studies comparing SSc to healthy controls have shown significantly impaired global longitudinal strain and global circumferential strain in SSc, prior to (and in the absence of) impairment in left ventricular ejection fraction [33, 34]. STE therefore offers potential and further studies are required; however, it remains limited by operator-dependency. Furthermore, echocardiography is unable to perform tissue characterization, which is central for SSc-myocardial disease.

#### Cardiac magnetic resonance

CMR has emerged as a central imaging tool for the detection of SSc myocardial involvement. CMR provides gold-standard, non-invasive imaging that characterizes the myocardium alongside highly sensitive and accurate evaluation of chamber size, function and morphology [35]. Cine SSFP (steady-state free precession) is employed to image the heart dynamically in real-time and quantifies cardiac size, structure and function, providing high resolution images to assess ejection fraction, ventricular volumes and wall motion abnormalities.

Tissue characterization techniques comprise a gadolinium-based contrast agent-derived T1 measure, late gadolinium enhancement (LGE), which is observed in the presence of myocardial necrosis, fibrosis and scarring. T2-weighted imaging is sensitive to water and allows qualitative detection of myocardial oedema and is usually compared with reference values from skeletal muscle. Quantitative mapping parameters are sensitive measures of the relaxation time of myocardial tissue (in milliseconds) that reflect tissue properties. These include (pre-contrast) T1 mapping, with high native T1 values observed with oedema (e.g. inflammation or infarction) as well as increased interstitial or extracellular space (as seen with fibrosis) and increased intracellular space (in hypertrophy). Post-contrast T1 mapping quantifies the extracellular space and pre- and post-contrast T1 calculates the extracellular volume (ECV) fraction. The ECV reflects the proportion of the myocardium that is extracellular that can be affected by oedema and fibrosis. As with T1 mapping, T2 mapping captures T2 relaxation times and is sensitive to oedema. Whilst tissue properties are a major attribute of CMR, it is fraught with technical challenges that have been extensively discussed [36].

Numerous studies have reported on CMR assessment of myocardial involvement in SSc, but these have been single centre, largely small observational reports and with different CMR protocols [5–7]. The pattern of LGE can provide insight into the aetiology of myocardial fibrosis, with subendocardial or transmural LGE typically reflecting ischaemic injury due to myocardial infarction, whereas in SSc-pHI, mid-wall or subepicardial myocardial LGE is characteristic. That said, multiple aetiologies can contribute to non-ischaemic LGE patterns (including hypertension and other co-morbidity) that should be considered. Furthermore, in an acute myocardial inflammatory setting, LGE may represent both oedema and necrosis that is partly reversible, while in chronic stages it is thought to primarily indicate the presence of (irreversible) scar. Quantitative T1 and T2 mapping studies have commonly identified presumptive diffuse myocardial fibrosis and/or inflammation. These findings have been reported in both asymptomatic and clinically presenting individuals with SSc, with prevalence estimates ranging from ∼40% to 70%. In a study of 83 SSc patients, we showed non-ischaemic LGE in 17/83 patients with significantly higher ECV, suggestive of diffuse fibrosis, with other studies reporting LGE even more commonly (up to 75% of cases) [5]. Poidron *et al.* showed abnormal native T1 in 50% of 72 patients with SSc [37] and Meloni *et al.* found abnormal native T1 and T2 in more than half of 55 patients with SSc [38].

Interpretation of CMR measures, however, notably T1 based LGE and T1 mapping, requires careful consideration in SARDs such as SSc. The chronic and fluctuating course of autoimmune disease, the more general inflammatory environment and the effect of background immunosuppression can influence CMR tissue characteristics. For this reason, the updated Lake Louise Criteria for myocarditis [39] that are now central to the updated ESC guidelines of inflammatory myopericardial syndromes [40] may not be sufficiently sensitive or specific in SSc. Studies in SSc and SARDs including SSc have been inconsistent. Also, the co-existence of ischaemic and non-ischaemic scar can contribute to increased signal on LGE images, as can secondary effects of PH and/or renal pathology.

CMR can also enable accurate quantitative perfusion mapping, with stress and rest myocardial blood flow measured and used to derive myocardial perfusion reserve as the stress-to-rest ratio akin to PET-CT (discussed later). Although no universally accepted thresholds have been defined, a global myocardial perfusion reserve below 2.0–2.5, in the absence of flow-limiting epicardial CAD and regional perfusion defects, is generally considered to support a diagnosis of CMD [41] however it is unclear whether this applies to SARD populations. Small to modest sized studies to date have suggested SSc patients show comparable rest myocardial blood flow (MBF) but reduced vasodilator stress MBF compared with healthy controls [8, 42, 43].

In general, CMR studies have been highly insightful but conflicting findings likely reflect the specific cohort evaluated (asymptomatic individuals *vs* those with clinical evidence of pHI) and limitations of modest sized cohorts and mainly cross-sectional evaluation. Large-scale longitudinal studies are lacking to inform on CMR’s ability to monitor myocardial disease and represent a critical knowledge gap to guide optimal clinical application.

#### Nuclear imaging: Positron emission tomography

Of the nuclear imaging techniques, SPECT is now of limited value, superseded by radiotracer-based PET-CT, although this is a more expensive and less accessible imaging modality. Only a small number of studies have evaluated PET for inflammation in SARDs. ^18^F-Fluorodeoxyglucose (FDG) PET-CT detects myocardial inflammation [44] but lacks specificity. Gallium-68 (^68^Ga)-fibroblast activation inhibitor (FAPI)-04A is a PET tracer that detects fibroblast activity and identifies active fibrotic processes in the heart. In a small proof of concept study, increased ^68^Ga-FAPI-04 tracer uptake was observed in six SSc patients with CMR-detected myocardial fibrosis compared with eight controls without fibrosis, with tracer activity higher in those with LGE, elevated NT pro-BNP and arrhythmia [45].

The non-invasive reference standard for detecting CMD is PET-CT, which allows fully quantitative assessment of MBF and myocardial flow reserve (MFR). A handful of myocardial perfusion studies employing the PET tracers rubidium-82 (^82^Rb) and nitrogen-13 (^13^N)-ammonia have been conducted in SARDs, including SSc. A first ^82^Rb PET study of RP that included patients with SSc reported substantial CMD (MFR < 2.0) in the SSc cohort and significantly reduced MFR in SSc-RP [1.62 (0.32)] compared with non-SSc SARD-RP [2.06 (0.61)] and healthy controls [2.22 (0.44)] [46]. The same group reported on a retrospective case–control study of 101 patients across several SARDs who underwent ^82^Rb PET and again demonstrated significantly reduced MFR, defined in this study as <1.5, compared with age-, sex- and comorbidity-matched patients without a SARD and more frequent reduced MFR (40% *vs* 22%) [47]. Reduced MFR in individuals with SARD was also associated with worse event-free survival when compared with patients with and without SARD and normal MFR and when compared with patients without SARD and low MFR.

### Electrocardiography and ambulatory monitoring

The 12-lead ECG provides an initial means of identifying electrophysiological abnormalities and is most reliable for detecting fixed conduction disturbances. Ambulatory Holter monitoring enables detection of supraventricular and ventricular arrhythmias and should be considered in symptomatic patients and/or when there is other evidence of SSc-pHI and a suspicion of myocardial involvement. Continuous monitoring with implantable loop recorder (ILR) may also be considered in patients at high risk of arrhythmia and/or when initial investigations have not identified the cause of symptoms. A pilot study using an ILR in 20 asymptomatic individuals with SSc detected significant arrhythmia including ventricular arrhythmia in almost half of participants [25].

#### Blood biomarkers

Whether and when to use advanced cardiac imaging for early detection, including in the subclinical setting and monitoring of SSc myocardial involvement, is unclear. Here, serum blood biomarkers may offer the opportunity for easy and accessible diagnostic test and/or means of stratifying for advanced imaging. Standard serum cardiac biomarkers, troponin (Tn), N-terminal pro-hormone BNP (NT-proBNP) and creatine kinase have been most widely evaluated. Cardiac Tn assays, however, may cross-react with skeletal muscle Tn [48] and therefore may be raised with skeletal muscle involvement and in the absence of cardiac involvement [49–51]. Studies have employed both cardiac TnI and TnT assays [37, 43], but TnI is thought to be more specific to the myocardium, with second generation high-sensitivity TnI assays now available that show no cross-reactivity to skeletal muscle Tn. NT-proBNP is established in the screening and prognostication of SSc-associated PAH [52, 53], and elevated NT-proBNP levels are associated with increased mortality in SSc [54]. A recent systematic review highlighted uncertainty regarding the role of Tn and NT-proBNP [55]. Their diagnostic utility is likely limited in early disease and confounded by known secondary effects of renal dysfunction and PH on both biomarkers; however, further studies are required to define the clinical settings in which they may have diagnostic value.

## Imaging, prognosis and outcomes

The value of imaging is also determined by its ability to provide prognostic information. Another recent limited systematic literature review examined the prognostic role of CMR measures on clinical outcomes, identifying nine studies ranging in cohort size from 24 to 260 individuals with SSc that described mainly myocardial rather than the multiple parameters of CMR [56]. Myocardial LGE, native T1 value, ECV and ventricular strain values appeared to predict adverse outcomes. In a cohort study of 150 patients with SSc and suspected cardiac involvement, myocardial to skeletal T2 ratio and LGE appeared to be predictors of ventricular arrhythmia, with the presence and extent of LGE alone the most predictive of ventricular arrhythmia [27]. In a study of 25 patients presenting with myocarditis, histopathological evidence on endomyocardial biopsy of higher grades of inflammation and fibrosis associated with higher event rate (defined as cardiovascular death, malignant arrhythmia needing implantable cardioverter defibrillator or rehospitalization due to heart failure) [1]. Sixteen of these individuals had CMR of which positive LGE was recorded in four (25%), all showing moderate to higher grade fibrosis on histology. In asymptomatic SSc patients, we have shown higher ECV to be associated with a trend toward poorer time-to-event outcomes, including future severe ventricular arrhythmia [25]. Higher values of T1-mapping and ECV have also been associated with future ventricular arrhythmic events [57].

Studies to date have been limited in nature and design. Large-scale studies of comprehensively phenotyped individuals with SSc are needed, using consistent definitions of CMR abnormalities and clearly defined adverse outcomes, to confidently identify imaging predictors of cardiac outcomes and survival and to clarify the relationship between specific myocardial tissue measures and clinical endpoints.

## Multimodality imaging approach

TTE is the first-line imaging modality employed when suspecting SSc pHI and is typically employed as part of annual monitoring for PAH in SSc. However, as discussed, it lacks sensitivity and specificity and, crucially, is unable to characterized myocardial tissue abnormalities that defines SSc-pHI. CMR is central to an early diagnosis if there is any clinical suspicion and has therefore become widely used in clinical practice and research studies. With challenges in the interpretation of CMR measures in SARDs such as SSc, PET may provide complementary value although studies to evaluate relative benefit are lacking. Both CMR and PET-CT have specific contraindications that may limit their use in individual patients. Table 1 summarizes the relative advantages and disadvantages of each imaging modality. When evaluating for myocardial disease, it remains essential to consider whether ischaemic heart disease and/or alternative aetiologies of cardiovascular involvement are the underlying pathology.

**Table 1:** Pros and cons of the main imaging modalities used to assess SSc primary heart involvement.

	Strengths	Limitations
TTE	Widely accessible, first line modality No radiation Myocardial strain and deformation analysis	Inability to characterize myocardial tissue Low sensitivity and specificity Suboptimal assessment of doppler signal for haemodynamic assessment Interobserver variability/operator dependent
CMR	No radiation Tissue characterization Superior spatial and temporal resolution (Quantitative) Perfusion assessment	Contraindications to contract agents Cost and limited accessibility Exclusion with severe renal insufficiency (gadolinium) Patient factors: claustrophobia or metallic implants Technical: susceptibility to image artifacts, limited temporal resolution, smaller field of view, specialist operator expertise and dependency in interpretation
PET	High sensitivity for inflammation Perfusion PET gold standard	High cost Radiation exposure Limited availability and access Inflammation assessment: ^18^F-FDG PET, non-specific (myocardial) ^18^F-FDG uptake and myocardial diet protocol Limited availability of perfusion PET

Multimodality imaging is often required to achieve diagnostic precision in SSc-pHI. CMR is the cornerstone of diagnosis, with PET used where additional clarification is required. When coronary artery atherosclerotic disease is suspected or diagnostic uncertainty persists, coronary CT angiography should be considered. Detailed here are the pros and cons of the usual imaging modalities for SSc-pHI. CMR: cardiovascular magnetic resonance; ^18^F-FDG: [^18^F]fluorodeoxyglucose; PET: positron emission tomography; pHI: primary heart involvement; SSc: systemic sclerosis; TTE: transthoracic echocardiography.

The World Scleroderma Foundation (WSF)-Heart Failure Association (HFA) consensus on screening, diagnosis, and follow-up assessment provides a first framework for application of CMR in clinical practice [4]. Screening of individuals considered at higher risk of myocardial involvement with CMR may be employed although practice varies, with interpretation in asymptomatic individuals and variable access leading to inconsistent use in this subgroup. Recommendations, including those from the British Society for Rheumatology systemic sclerosis guidelines [58] and an early UK SSc–Cardiac consensus group [59], provide further guidance on the broader use of cardiovascular investigations.

## Treatment of SSc-pHI

Two broad therapeutic strategies may be considered for SSc-pHI, specific disease-targeted treatment of SSc myocardial involvement and guideline-directed medical therapy (GDMT) based on contemporary cardiovascular and heart failure management.

To date, no therapies have been specifically tested for SSc myocardial involvement. Current management largely extrapolates from immunosuppressive strategies used for SSc and major organ involvement, particularly ILD. Observational studies and case series suggest potential benefit in both subclinical and overt cardiac involvement with DMARDs including mycophenolate mofetil, rituximab, tocilizumab and pulsed cyclophosphamide [60]. In addition, preliminary proof-of-concept data indicate that antifibrotic therapies developed for SSc-associated ILD may favourably influence CMR-derived measures of myocardial fibrosis. Glucocorticoids, which are commonly used in other forms of SARD-associated myocarditis, should be used with caution in SSc because of the increased risk of scleroderma renal crisis. Whether targeting the underlying microvasculopathy, using established or novel vasodilator therapies, can modify the trajectory of myocardial disease is plausible but yet to be tested.

Addressing these questions requires deeper mechanistic insight alongside robust, disease-specific trial designs. Given the low absolute incidence of hard clinical endpoints in SSc, CMR could provide an important opportunity to develop and validate sensitive imaging biomarkers as surrogate trial endpoints. However, the technical challenges of CMR described earlier pose significant obstacles to its application in multicentre studies. Also, dedicated intervention trials with myocardial disease as a primary end point are unlikely to be feasible in SSc; instead, basket trial designs, analogous to those employed in SARD-ILD, may represent a more pragmatic approach. Meanwhile, systematic capture of clinical cardiac events in ongoing and future trials, together with the incorporation of sensitive CMR measures as secondary endpoints, if current technical challenges can be addressed, would generate meaningful cumulative insights into myocardial disease modification.

In addition to immunosuppressive strategies, close multidisciplinary collaboration with cardiology specialists is essential to ensure timely implementation of GDMT. Contemporary heart failure treatments, such as sodium–glucose cotransporter-2 inhibitors, now form a cornerstone of management and should be considered in SSc based on standard guidance. However, their efficacy and safety have not been specifically evaluated in SARDs, including SSc. Moreover, the early detection of SSc myocardial involvement in the absence of overt heart failure offers a potential window for intervention at a stage when disease progression may still be modifiable, with the aim of preventing or delaying the development of heart failure. Notwithstanding the limitations of conducting interventional trials discussed earlier, the use of GDMT should ideally move beyond extrapolation from the general population and instead define the optimal timing, selection, and integration of established and emerging therapies within SSc-specific disease pathways.

**Figure 2:**
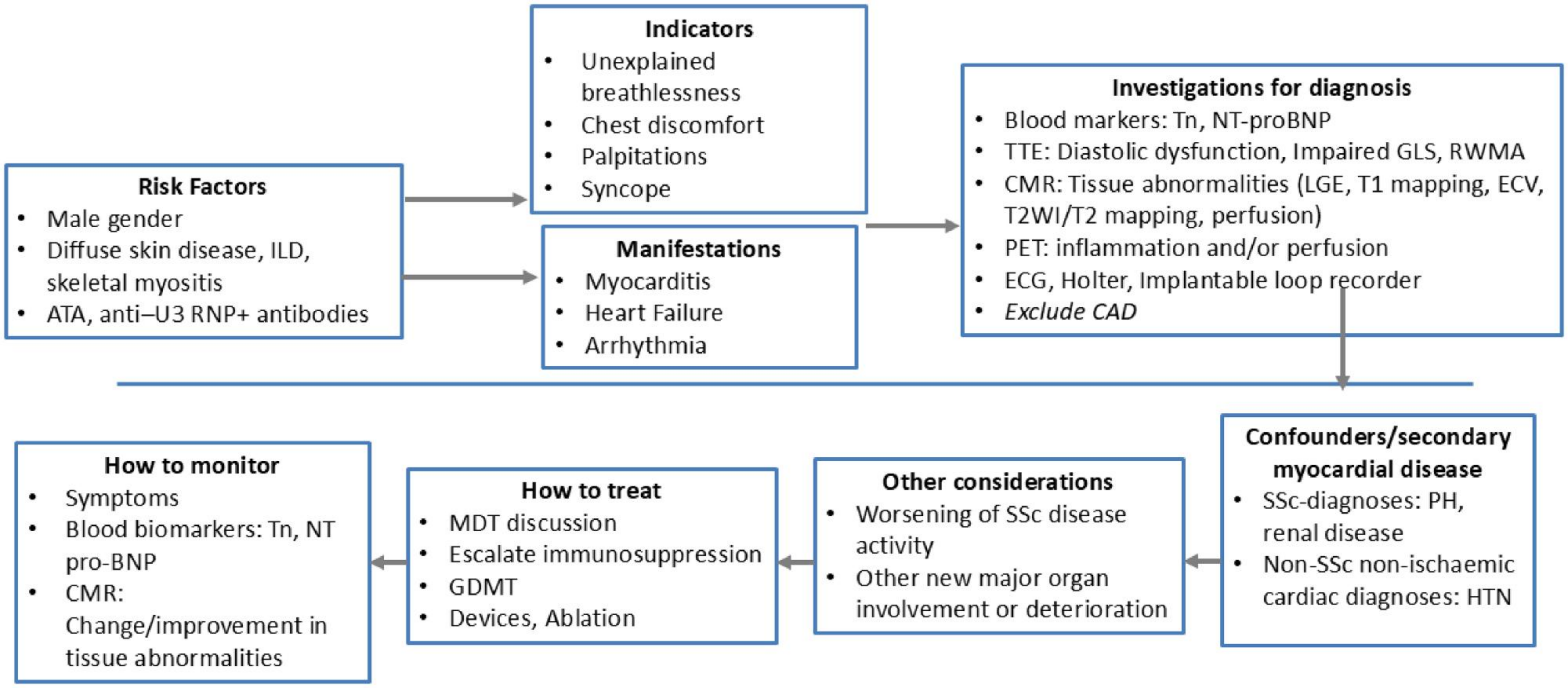
Considerations in the management of SSc primary heart involvement. Schematic overview of the considerations in the management of systemic sclerosis–primary heart involvement (SSc-pHI). Key risk factors include autoantibody profile [anti-topoisomerase I (ATA), anti-U3 RNP], diffuse cutaneous disease, interstitial lung disease and skeletal myositis. Clinical manifestations include myocardial inflammation and arrhythmia, although cardiac involvement may be subclinical. Circulating biomarkers of myocardial injury (troponin) and ventricular stress (NT-proBNP) may be elevated but can be normal, particularly in subclinical disease. Echocardiography is typically first-line; however, CMR is central to diagnosis, with PET considered when CMR is not feasible or findings are indeterminate. Arrhythmic risk assessment should include ECG, with ambulatory monitoring or implantable loop recorders considered where suspicion persists. No disease-specific therapy exists for SSc-pHI; management involves optimization of guideline-directed cardiovascular therapy and escalation of systemic sclerosis immunosuppression, underscoring the importance of multidisciplinary cardiology–rheumatology care. ATA: anti-topoisomerase I; CAD: coronary artery disease; CMR: cardiovascular magnetic resonance; ECV: extracellular volume; GDMT: guideline directed medical therapy; GLS: global longitudinal strain; ILD: interstitial lung disease; LGE: late gadolinium enhancement; MDT: multi-disciplinary team; NT-proBNP: N-terminal pro-B-type natriuretic peptide; PET: positron emission tomography; PH: pulmonary hypertension; RNP: ribonucleoprotein; RWMA: regional wall motion abnormality; SSc: systemic sclerosis; T2WI: T2 weighted imaging; Tn: troponin; TTE: transthoracic echocardiography

Figure 2 illustrates the considerations in the management of SSc primary heart involvement.

## Future directions

The WSF/HFA consensus guidance on the screening, diagnosis and follow-up assessments for SSc-pHI [4] provides a first framework for a standardized algorithm to introduce a consistent approach in related research and clinical practice. However, multicentre, longitudinal studies are needed to support stronger evidence-based screening and monitoring strategies using advanced cardiovascular imaging. The development of surrogate trial endpoints would represent a significant step to enable testing of specific therapies for SSc-pHI, but considerable challenges remain. In addition, mechanistic understanding through experimental study is needed for discovery of tractable therapeutic targets.

## Conclusion

**Figure 3:**
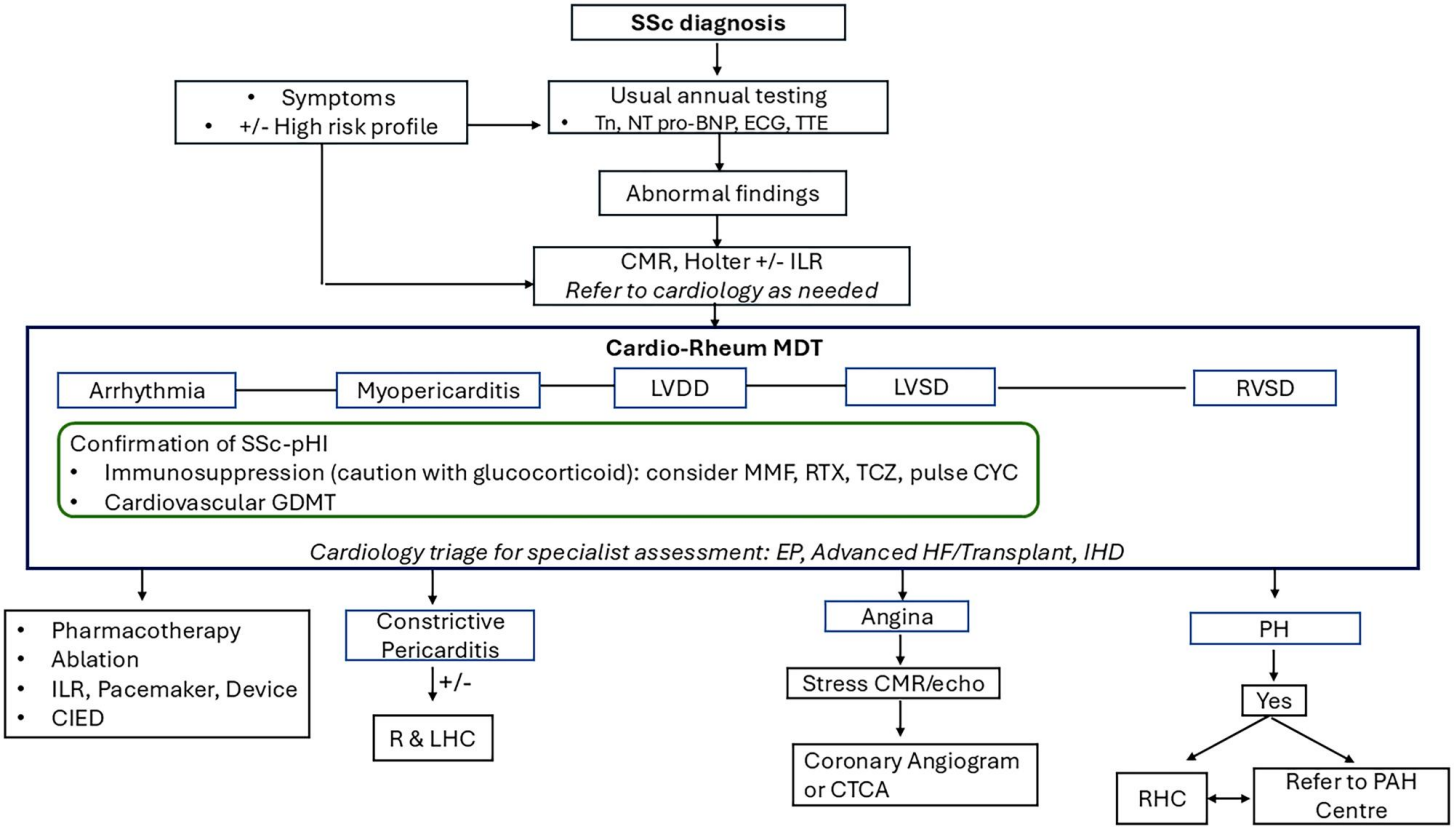
Framework for managing SSc primary heart involvement. A multidisciplinary approach involving close collaboration with cardiology is important to enable accurate diagnosis and timely intervention, including initiation of both immunosuppressive therapy and guideline-directed cardiac treatments where indicated. Current consensus supports annual measurement of Tn and NT-proBNP alongside ECG and TTE, typically undertaken as part of PAH monitoring. Symptoms not attributable to ILD or PAH, and/or abnormalities on routine investigations, should prompt referral for CMR. CMR may also be considered in individuals asymptomatic for cardiac involvement but with a high-risk profile for SSc-pHI—such as cutaneous subtype, SSc-specific autoantibodies, and features of active disease; however, access limitations mean this is usually assessed on a case-by-case basis. Where myocardial involvement is identified, secondary causes related to pulmonary vascular and renal pathology should be considered, alongside alternative non-SSc cardiovascular conditions. Depending on the results of these investigations, further diagnostic assessment and referral to specialist services may be required including advanced HF service and management of arrhythmia. Additional findings of non-SSc cardiovascular disease, such as left ventricular systolic dysfunction, should prompt evaluation for coronary artery disease and other relevant cardiac pathology. CAD: coronary artery disease; CIED: cardiac implantable electronic device; CTCA: computed tomography coronary angiogram; CMR: cardiovascular magnetic resonance; CYC: cyclophosphamide; EP: Electrophysiology; GDMT: guideline directed medical therapy; HF: heart failure; IHD: ischaemic heart disease; ILR: implantable loop recorder; LVDD: left ventricular diastolic dysfunction; LVSD: left ventricular systolic dysfunction; MDT: multi-disciplinary team; MMF; mycophenolate mofetil; NT-proBNP: N-terminal pro-B-type natriuretic peptide; PET: positron emission tomography; PH: pulmonary hypertension; pHI: primary heart involvement; PAH: pulmonary artery hypertension; R & LHC: right and left heart catheterization; RTX: rituximab; RVSD: right ventricular systolic dysfunction; SSc: systemic sclerosis; TCZ: tocilizumab; Tn: troponin; TTE: transthoracic echocardiography

SSc carries one of the highest cardiovascular risks among autoimmune diseases, with primary myocardial disease underpinning this excess morbidity and mortality. Clinical presentations are heterogeneous and comprise myocarditis, heart failure, serious arrhythmia and sudden cardiac death, emphasizing the need for early detection. Advanced cardiac imaging, primarily CMR with PET as a complementary modality, plays a central role in identifying subclinical and confirming overt myocardial disease but needs careful assessment for precise interpretation. A multidisciplinary team working with cardiology and/or cardiac radiology is essential to provide accurate diagnosis and timely initiation of both immunosuppressive and guideline directed cardiac therapies (Fig. 3). Systematic assessment for SSc-pHI should be integrated into routine SSc care to improve quality of life and outcomes of people with SSc. Concerted basic and translational research programmes are needed to address the knowledge gaps in diagnosis and monitoring and enable the development of therapies specifically targeting SSc myocardial disease. To this end, initiatives such as the UK CARDIO-IMID Partnership provide a promising platform (https://ukcardioimid.co.uk/).

## Data Availability

No new data were generated or analysed in support of this article.
